# The implications of Paulo Freire’s critical aesthetics for individual mental health

**DOI:** 10.3389/fpsyg.2026.1813138

**Published:** 2026-07-29

**Authors:** Ming-Kuo Chen, Yi-Huang Shih

**Affiliations:** 1 Department of Insurance and Finance Management, Chaoyang University of Technology, Taichung, Taiwan; 2 Center of Teacher Education, Minghsin University of Science and Technology, Xinfeng, Hsinchu, Taiwan

**Keywords:** critical aesthetics, dialogue, hope, mental health, older adults, Paulo Freire

## Abstract

This article seeks to explore Paulo Freire’s concept of critical aesthetics and to further interpret its implications for promoting individual mental health. In Paulo Freire’s conception of critical aesthetics, mental health should not be understood as mere adaptation to existing social realities. Rather, it signifies the restoration of the human being as a speaking, imagining, hoping, and meaning-making subject. From this perspective, mental health refers to the individual’s reconstitution as an autonomous and agentic subject—one who not only regains a sense of selfhood, but also embraces life with hope and engages in purposeful, transformative praxis within the world.

## 1 Introduction

Mental health disorders represent a pressing global public health issue, imposing a substantial burden on individuals, families, and societal and economic development. According to the World Health Organization (WHO), mental disorders are among the leading contributors to the global disease burden. These conditions severely disrupt daily life, occupational functioning, and interpersonal relationships. For instance, major depressive disorder often manifests as persistent low mood, loss of interest, and fatigue, significantly impairing an individual’s ability to study or work (World Health Organization, 2022; Yang et al., 2026; Seligman and Csikszentmihalyi, 2000). Research has suggested that experiences of social exclusion, marginalization, misrecognition, and a lack of social support are associated with increased risks of depression, anxiety, and reduced psychological well-being (Honneth, 1996; Keyes, 2002; Ryff and Singer, 2008). From this perspective, recognition may be understood as an important psychosocial condition that contributes to individuals’ mental health and flourishing. Therefore, mental health disorders should not be regarded merely as individual health concerns but as significant social issues that affect collective well-being and sustainable development. Addressing these challenges through effective prevention measures, educational promotion, support systems, and intervention strategies has become a critical priority for governments, healthcare professionals, and educators worldwide. Consequently, exploring theoretical and practical approaches that promote mental health and enhance individual well-being is of considerable importance for both academic research and professional practice. Furthermore, increasing attention has been directed toward identifying innovative and insightful educational and philosophical approaches to addressing mental health challenges. In this context, Paulo Freire’s critical aesthetics offers valuable theoretical insights. The core principles emphasized in Freire’s critical aesthetics—including self-awareness, empowerment, dialogue, hope, and humanization—not only contribute to the development of critical consciousness but also promote individual mental health and overall well-being (Freire, 1975, 1994, 2000; World Health Organization, 2022).

Freire (1921–1997) was a renowned Brazilian educator, educational philosopher, and one of the foremost pioneers of Critical Pedagogy. His educational thought and practice not only profoundly influenced educational reform in Brazil but also gradually extended throughout Latin America and across the globe, exerting a significant impact on educational development, social emancipation, and the advancement of democratic processes (Shaull, 2000). Owing to his substantial contributions to educational theory and practice, Freire is widely regarded as one of the most influential educational thinkers of the twentieth century. His work continues to serve as a foundational theoretical framework for contemporary critical pedagogy.

The formation of Freire’s educational philosophy was closely intertwined with the socio-historical conditions in which he lived. Growing up in a Brazilian society characterized by severe economic disparities and structural inequalities, Freire developed a profound awareness of the limitations that poverty, oppression, and social exclusion impose upon human development. Consequently, he viewed education as a vital means of personal liberation and social transformation. He argued that education should empower oppressed individuals to develop critical consciousness and actively engage in social reform and democratic participation (Freire, 1997, 2000). According to Gadotti (1994), Freire can be understood as an “organic intellectual,” a concept originally proposed by the Italian philosopher Antonio Gramsci. An organic intellectual is not merely a producer of knowledge but also an individual who assumes the responsibility of responding to social realities and promoting social change. Freire’s educational philosophy was grounded in a profound critique of social injustice and oppression, reflecting a deep concern for marginalized populations and a strong commitment to social justice, democratic participation, and human emancipation (Shaull, 2000). Because of both its theoretical depth and practical significance, Freire’s educational thought has received widespread recognition within the international academic and educational communities. In 1986, *The New York Times* published an article highlighting the influence of Freire’s ideas on educational reforms in various countries around the world (Gadotti, 1994). To this day, the core concepts advocated by Freire—including dialogue, critical reflection, praxis, and humanizing education—continue to shape contemporary educational reform and pedagogical innovation. His work remains an important theoretical resource for advancing discussions of democratic education, citizenship education, and educational justice.

Although Paulo Freire did not develop a comprehensive and systematic theoretical framework explicitly under the concept of *aesthetics*, his works, including *Pedagogy of the Oppressed*, *Cultural Action for Freedom*, and *Pedagogy of Hope*, articulate ideas closely related to liberation, dialogue, humanization, and social transformation. Within Freire’s educational philosophy, aesthetics encompasses more than perception, creativity, and expression; it is fundamentally concerned with how individuals develop critical consciousness, reinterpret their relationships with themselves and the world, and pursue humanization and freedom through transformative praxis (Freire, 1994, 1997, 2000).

In recent years, research on mental health has increasingly emphasized the influence of factors such as meaning in life, subjectivity, autonomy, social connectedness, and self-actualization on psychological well-being (Ryan and Deci, 2001; Ryff, 1989a, 1989b). Moreover, human flourishing has been recognized as an important objective that extends beyond the prevention of illness and the alleviation of psychological symptoms. At its core, human flourishing involves the realization of individual potential, active social participation, and the cultivation of a meaningful life (Keyes, 2002; Seligman, 2011). However, existing studies on aesthetics and mental health have largely focused on the effects of artistic participation, aesthetic experiences, or art therapy on psychological outcomes (Fancourt and Finn, 2019). Comparatively little attention has been given to examining how aesthetics, from the perspective of critical pedagogy, may serve as a significant pathway for promoting psychological well-being and human development.

On the other hand, existing studies on Paulo Freire’s thought have primarily focused on issues such as critical pedagogy, social justice, emancipatory education, and democratic participation (Giroux, 2010; McLaren, 2015; Shih, 2026). In comparison, relatively little attention has been devoted to examining the theoretical implications of his concept of critical aesthetics and its potential relevance to mental health. In recent years, mental health research has increasingly shifted beyond a disease-oriented perspective to emphasize psychological well-being, including individuals’ sense of meaning, subjectivity, autonomy, social connectedness, and self-realization. However, the potential contributions of Freire’s critical aesthetics to these dimensions of mental health remain largely underexplored. Specifically, insufficient attention has been given to how critical aesthetics may foster psychological well-being and human flourishing through the development of critical consciousness, the construction of subjectivity, and creative engagement with social reality. Therefore, the relationship between critical aesthetics and mental health warrants further theoretical investigation. To address this gap, this article aims to explore the core concepts and theoretical foundations of Freire’s critical aesthetics and to examine their implications for individual mental health. More specifically, the study analyzes how the central principles of critical aesthetics may contribute to the development of subjectivity, psychological well-being, and human flourishing, while also considering the broader implications of Freire’s thought for contemporary mental health promotion and educational practice. Based on the research gap identified above, this article seeks to address the following research questions:

1. What are the central concepts and theoretical underpinnings of Freire’s critical aesthetics?
2. In what ways can Freire’s critical aesthetics contribute to the development of psychological well-being and human flourishing?
3. What theoretical insights and practical implications can Freire’s critical aesthetics provide for contemporary mental health education and educational practice?

## 2 Methodology

This study adopts critical hermeneutics as its primary methodological approach to examine the core concepts of Paulo Freire’s critical aesthetics and their theoretical implications for mental health and human flourishing. Critical hermeneutics integrates the traditions of critical theory and hermeneutics, emphasizing that researchers should not only seek to understand the meanings conveyed within texts but also critically reflect upon the historical, cultural, social, and power structures embedded within them. Through such reflection, critical hermeneutics aims to uncover the potential contributions of these texts to human emancipation and social transformation (Habermas, 1984; Ricoeur, 1976).

A closer examination of Freire’s theory of conscientization reveals that he consistently regarded critique as a fundamental means through which human beings pursue freedom and liberation. Freire argued that individuals are not passive recipients of a predetermined reality; rather, through critical reflection and praxis, they actively engage with their lifeworld and, in doing so, transform both themselves and the social conditions in which they live (Freire, 2000; Gadotti, 1994). Consequently, critique constitutes not only a central pillar of Freire’s educational philosophy but also a crucial theoretical foundation for understanding his conception of critical aesthetics. From a theoretical perspective, critical hermeneutics can be understood through two interrelated dimensions: critique and hermeneutics. First, with regard to *critique*, its intellectual roots can be traced to Immanuel Kant’s critical philosophy. The purpose of critique is to examine whether the knowledge, beliefs, and values accepted by individuals are rationally justified and legitimate, while employing reason, judgment, and reflection to reveal the potential limitations embedded within prevailing ideas and social structures. Second, with regard to *hermeneutics*, understanding constitutes its central concept. Hermeneutics maintains that all understanding is grounded in specific historical, cultural, and social contexts; therefore, there can be no entirely objective knowledge or universal interpretation that exists independently of context (Gadamer, 2004). When critique and hermeneutics are brought together, critical hermeneutics maintains that researchers, in interpreting texts and ideas, should not only examine their surface meanings but also critically reflect on the historical conditions, social contexts, and value orientations that have shaped them. At the same time, researchers must avoid absolutizing or universalizing any particular interpretation, since individuals may arrive at different understandings and interpretations as a result of their diverse life experiences and social contexts. Based on this perspective, the present study employs a critical hermeneutic approach to analyze Freire’s major works, including *Pedagogy of the Oppressed*, *Cultural Action for Freedom*, and *Pedagogy of Hope*. These texts are examined in dialogue with contemporary literature on mental health, psychological well-being, and human flourishing through critical interpretation and theoretical engagement, with the aim of elucidating the core meanings of Freire’s critical aesthetics and its implications for contemporary mental health education and educational practice.

## 3 The interpretation of Paulo Freire’s critical aesthetics

### 3.1 Human beings are beings of subjectivity

Paulo Freire sought to rediscover the humanizing vocation of intellectuals and, through the power of critical thought, to negate existing limitations and point toward a renewed future for humanity. The reason Freire was able to envision such an ideal lies in his fundamental ontological assumption: the ontological vocation of human beings is to become Subjects capable of acting upon and transforming the world in which they live. Only by doing so can individuals manifest their subjectivity, enrich both individual and collective life, open up new possibilities, and ultimately become authors of their own history. From an existential perspective, the world to which human beings are connected is neither static nor a closed order. Likewise, those who live within it are not merely destined to accept a given reality and adapt themselves to it. On the contrary, human beings must hold the conviction that they are Subjects rather than objects (Freire, 1997, 2000; Shaull, 2000). That is, they must believe that they are Subjects of the world. This conviction indirectly reveals that human beings possess subjectivity and autonomy; they are beings endowed with agency. Thus, Freire (1997) asserts that within the world in which humans exist—and even within the interaction between humans and the world—the proper role of the human being is that of Subject. Furthermore, Freire emphasizes that human beings not only live in the world but are capable of entering into relationships with it, participating in the ongoing formation and transformation of their existential reality. In their interaction with the world, humans are Subjects rather than objects; they are capable of envisioning and planning their own future. Therefore, the human role in the world is not passive. Human beings create the world and are capable of changing it. They possess critical capacities that enable them to choose, transform, and reconstruct reality. Accordingly, those who achieve critical consciousness come to recognize themselves as Subjects of the world—beings endowed with subjectivity. Human beings are thus active agents of knowing and acting, rather than objects to be known or passively shaped by external forces. Moreover, Freire argues that a fundamental condition of conscientization is precisely this recognition of oneself as Subject. Only when individuals become aware that they are Subjects rather than objects does the possibility of critical consciousness emerge. Only then can the individual become a mode of existence characterized by subjectivity.” (Freire, 1975, 1985, 1997, 2000).

### 3.2 Dialogue as a foundation for liberation and critical education

Paulo Freire was raised in a Catholic family and gradually developed a form of Catholic thought characterized by democratic radicalism and a counter-hegemonic orientation during his youth. This intellectual perspective later became a significant foundation of his educational philosophy. Freire firmly believed that human beings possess the capacity to transform their lived world. When individuals find themselves situated within unjust social realities, social transformation becomes possible if they critically recognize the irrational and oppressive nature of those realities and engage in authentic praxis. Driven by this conviction, Freire actively participated in Brazil’s adult literacy movement. Through literacy education, he sought not only to develop the reading and writing abilities of illiterate adults but also to realize the ideals of liberatory education. He aimed to enable learners to understand the oppressive structures embedded within their lived world and to cultivate the capacity and courage necessary to transform unjust social conditions. In doing so, learners could develop their subjectivity, autonomy, and critical consciousness. Within this process, dialogue occupies a central position. For Freire, dialogue is not merely a pedagogical method but also the fundamental basis of liberatory and critical education. Through dialogue, teachers and students are able to read the social reality critically, reflect upon social issues, and achieve humanization through relationships grounded in mutual respect and understanding. Therefore, dialogue serves not only as a crucial pathway for cultivating critical consciousness but also as an essential foundation for individual liberation and human development (Elias, 1976; Freire, 1997, 2000; Shih, 2026).

Freire’s philosophy of dialogue as a foundation for liberation and critical education. Freire defines dialogue as a human encounter mediated by the world. This means people do not simply talk to one another; they engage together in critically interpreting reality. “Naming the world” refers to developing critical consciousness — the ability to recognize social, political, and economic structures and to understand one’s position within them. Dialogue therefore becomes a collaborative act of meaning-making and world-creation. Because dialogue is rooted in shared human agency, it cannot exist where one party denies the other’s right to speak or interpret reality. If one group claims exclusive authority to define truth while others are expected only to accept it, dialogue collapses into oppression. For Freire, every person has the ontological and political right to name the world. Denying this right dehumanizes both the oppressed and the oppressor. Freire also rejects what he calls the “banking model” of education, where knowledge is “deposited” by teachers into passive students. In such a model, learners are objects rather than subjects of knowledge. Genuine dialogue, by contrast, positions all participants as co-creators of understanding. Knowledge is not transferred; it is produced through reflection and action (praxis) finally; Freire insists that dialogue cannot be neutral. It either reinforces domination or challenges it. Authentic dialogue resists domination because it is grounded in humility, mutual respect, love, hope, and critical thinking. It seeks transformation rather than control. In this sense, dialogue becomes both a method of education and a practice of freedom (Chen et al., 2025; Freire, 1997, 2000; Shih, 2018a, 2018b, 2026).

### 3.3 Hope as praxis

Freire believed that hope is not merely an emotion or a fantasy, but is rooted in humanity’s desire and capacity to transform the world. Based on this life experience, Freire proposed the concept of the Pedagogy of Hope. He argued that education should be a critical and practice-oriented process, enabling the oppressed to reimagine the future and take action to change their reality hope as praxis (Freire, 1994, 2000). The Pedagogy of Hope is not a naive or optimistic expression, but a morally grounded practice with agency, with hope rooted in the openness of history and humanity’s potential for transformation. Through ongoing dialogue and processes of conscientization in education, Freire encouraged learners not only to accept reality but to become “co-creators of reality.” Indeed, Freire’s interpretation and extension of hope, discussed through the transformative relationship between the oppressors and the oppressed, emphasizes key concepts such as hope, love, dialogue, and critical consciousness. Freire’s life exemplified a profound passion for life, a passion rooted in love and sustained by hope. From the economic hardship of his childhood, to witnessing social injustices in his youth, and later dedicating himself to the movement for liberatory education, Freire transformed his personal life trajectory into a practice of educational philosophy—highlighting the inseparable relationship among hope, love, dialogue, and critical consciousness (Freire, 1994).

The core values emphasized by Freire—including hope, subjectivity, agency, love, dignity, and freedom—are closely related to individual mental health, psychological well-being, and human flourishing. Human beings are not inherently inclined toward either active engagement or passive alienation; rather, their orientations are shaped largely by the sociocultural contexts and living conditions in which they develop and function. When individuals are situated within environments that support autonomy, dignity, and social participation, they are more likely to demonstrate subjectivity and agency, enabling them to actively interpret and respond to their lived circumstances. As a result, they can develop a stronger sense of self-efficacy, autonomy, and meaning in life. Conversely, prolonged exposure to environments characterized by oppression, domination, and alienation may lead individuals to become passive, disengaged, and even psychologically distressed (Freire, 1997, 2000; Ryan and Deci, 2000). From Freire’s perspective, hope is not merely an individual emotion or optimistic attitude; rather, it is a critical and transformative force grounded in human praxis. Hope empowers individuals to imagine alternative possibilities, challenge oppressive realities, and participate actively in the transformation of society. In this sense, hope functions as an essential psychological and existential resource that supports resilience, meaning-making, and personal growth. Consequently, Freire’s conception of hope bears significant relevance to contemporary discussions of psychological well-being and human flourishing (Freire, 1994).

Moreover, hope, love, and dignity contribute to the development of positive interpersonal relationships, strengthen psychological resilience, and promote emotional health and overall well-being (Ryff, 1989a, 1989b; Snyder, 2002). In addition, freedom signifies more than the absence of external oppression and constraints; it represents individuals’ capacity to make autonomous choices, realize their intrinsic worth, and pursue meaningful and fulfilling lives. Through the exercise of freedom, individuals are able to humanize their lifeworld and actively participate in shaping a more hopeful future (Freire, 2000). Therefore, these values are not only central components of Freire’s emancipatory pedagogy and critical aesthetics but also provide an important theoretical foundation for promoting mental health, enhancing psychological well-being, and fostering human flourishing. From this perspective, Freire’s critical aesthetics extends beyond educational theory and offers valuable insights into contemporary discussions of mental health by emphasizing the importance of hope, dignity, agency, freedom, and humanization in supporting individuals’ psychological growth and overall flourishing (Freire, 1994, 2000; Keyes, 2002; Ryff, 1989a, 1989b; Seligman, 2011; Snyder, 2002).

## 4 The interpretation of how Paulo Freire’s critical aesthetics promotes individual mental health

### 4.1 Mental health is the individual’s sense of being restored as an autonomous subject

There is growing recognition in the fields of public health and mental health services research that the provision of clinical services to individuals is not a viable approach to meeting the mental health needs of a population. Mental health can be understood as the individual’s sense of being restored as an autonomous subject. Psychological distress is not merely a matter of personal pathology but represents an existential state closely linked to oppression, silencing, voicelessness, and devaluation. Experiences of depression, anxiety, and helplessness often emerge from being denied recognition of one’s value and existence. Psychological recovery, therefore, begins with experiences in which the individual feels seen, respected, and understood. Freire’s critical aesthetics provides a framework for understanding mental health as the restoration of subjectivity through creative and dialogical processes. Critical consciousness (conscientization) allows individuals to recognize structural sources of oppression while simultaneously engaging in aesthetic and creative practices that facilitate self-expression and emotional integration. For instance, art-based interventions and participatory creative activities have been shown to enhance emotional regulation, self-efficacy, and social connectedness, all of which contribute to improved mental health outcomes (Deci and Ryan, 2008; Eisner, 2002; Freire, 1994, 2000; Hooks, 1994; Kabat-Zinn, 2003; Malchiodi, 2012; Purtle et al., 2020). From this perspective, promoting mental health requires more than individual therapy; it necessitates creating contexts in which individuals can be seen and heard, critically reflect on their social conditions, and engage in creative expression and dialogical interaction. Such an approach underscores the inseparability of social justice, aesthetic engagement, and psychological well-being, illustrating that mental health is fundamentally about reclaiming one’s subjectivity in the face of social and structural constraints (Freire, 1994; Hooks, 1994).

### 4.2 Healing and restoration in dialogical relationships: co-creating the meaning of life

As Paulo Freire emphasizes, dialogue is not merely a linguistic exchange or a communicative technique; rather, it is a process of shared understanding and critical reflection upon reality. Dialogue is a historical and praxical act through which people collectively interpret and “name” the world they inhabit. To “name the world” is not to describe it neutrally, but to engage in a critical understanding of social, political, and economic structures, to identify oppression and injustice within them, and to acquire the subjectivity necessary to speak and transform reality. In this sense, dialogue is not an instrumental method but an ethical and aesthetic practice. Its ethical dimension lies in the recognition of the dignity and subjectivity of the other; its aesthetic dimension lies in the creation of meaning and the unfolding of human possibility. Dialogical relationships thus become generative spaces—spaces in which participants, through mutual listening, response, and shared reflection, reconstruct wounded self-narratives, restore silenced voices, and reaffirm their existence as acting subjects. From the perspective of critical aesthetics, dialogue does more than expose reality; it opens the horizon of imagination. It enables individuals to reposition themselves within their historical context, transforming fragmented experiences into meaningful, articulable, and actionable structures of understanding. Within such dialogical relationships, healing is not merely psychological adjustment, but a process through which life is reconnected to hope and possibility through shared critical consciousness and transformative action. Therefore, Freire’s conception of dialogue reveals a profound educational implication: education is not the transmission of knowledge, but the generation of new meaning through ethical recognition and aesthetic creation. Dialogue itself becomes a site of restoration and healing, allowing life to manifest its unfinished and open character through co-creation, and to move continuously toward more humanizing modes of existence (Freire, 1994, 1997, 1998, 2000).

### 4.3 Embracing life with hope and engaging in purposeful, transformative action

In *Pedagogy of Hope*, Freire (1994) conceptualized hope not as passive waiting or sentimental optimism, but as a practical force grounded in humanity’s capacity to transform the world—hope as praxis. For Freire, hope must be dialectically linked with critical consciousness (conscientização) and transformative action (praxis). Without action, hope deteriorates into naïve illusion; without hope, action devolves into cynicism and despair (Freire, 1994, 2000). Hope, therefore, is neither psychological comfort nor abstract belief, but an ontological and ethical stance toward history as open and unfinished. From a mental health perspective, this conception provides profound implications. Mental health should not be narrowly defined as the absence of psychological symptoms or emotional instability. Instead, it may be understood as the individual’s capacity to imagine alternative futures and to believe in their agency to participate in change—even while situated within suffering. Contemporary psychological research supports this interpretation. Snyder’s (2002) hope theory conceptualizes hope as involving both agency (goal-directed determination) and pathways (planning to meet goals), both of which are associated with resilience and psychological well-being. Similarly, Bandura (1997) demonstrated that self-efficacy—the belief in one’s capacity to influence outcomes—significantly predicts adaptive coping under stress. Freire’s notion of hope as praxis resonates with these findings, as it strengthens agency, meaning-making, and action orientation in the face of adversity.

When hope is stripped away, time becomes psychologically frozen in the traumatic moment, and the individual’s narrative continuity is disrupted. Freire’s critical aesthetics operates precisely at this existential level: by reopening imagination and possibility, it restores the subject’s relationship to the “not yet” (Freire, 1994). Through dialogical engagement and creative expression, individuals may reconstruct meaning and reposition themselves not merely as victims of history, but as participants in its transformation. Mental health, therefore, may be reconceptualized as a temporal and ethical unfolding. It is not simply emotional equilibrium, but the capacity to project oneself toward the future, to inhabit history as unfinished, and to act within that openness. In Freirean terms, psychological well-being involves reclaiming oneself as a historical subject—one who does not merely endure reality, but engages in its transformation.

## 5 Reflections

### 5.1 Strengthening the manuscript’s theoretical integration and internal coherence

Although Freire’s critical aesthetics was not primarily developed as a framework for addressing mental health, its emphasis on humanization, dialogue, critical consciousness, and liberation bears significant theoretical relevance to contemporary perspectives on psychological well-being and human flourishing. First, The Psychological Well-Being Scale (Ryff, 1989b) is intended to measure aspects of positive functioning. Ryff’s (1989a) theory of psychological well-being identifies autonomy, environmental mastery, personal growth, positive relations with others, purpose in life, and self-acceptance as essential dimensions of mental health. These dimensions are highly consistent with Freire’s conception of humanizing education, as the process of humanization seeks to foster individuals who are autonomous, critically reflective, and capable of self-realization. Through the development of critical consciousness and active engagement with the world, individuals are empowered to transcend passive adaptation and become agents of their own growth and transformation. Consequently, Freire’s vision of humanization provides a meaningful educational foundation for promoting psychological well-being and supporting the development of a flourishing life.

Second, human beings can be proactive and engaged or, alternatively, passive and alienated, largely as a function of the social conditions in which they develop and function (Ryan and Deci, 2001). Ryan and Deci’s (2001) Self-Determination Theory (SDT) posits that autonomy, competence, and relatedness are three fundamental psychological needs essential for promoting mental health and well-being. Freire’s emphasis on dialogical relationships and collaborative inquiry not only fosters learners’ sense of agency and subjectivity but also strengthens their connections with others and the broader social community. Through meaningful dialogue and participatory learning, individuals are encouraged to actively engage in the construction of knowledge, develop confidence in their abilities, and establish authentic relationships with others. In this regard, Freire’s dialogical pedagogy contributes to the fulfillment of the psychological needs identified by Self-Determination Theory, (Deci and Ryan, 2008) thereby supporting psychological well-being and human development.

Furthermore, Honneth’s (1996) Recognition Theory argues that an individual’s sense of identity and psychological well-being depends upon receiving respect, care, and social recognition from others. When individuals experience prolonged conditions of neglect, oppression, or exclusion, they are more likely to develop self-denial, diminished self-worth, and psychological distress. This perspective closely resonates with Freire’s analysis of the condition of the oppressed. Freire maintained that education should facilitate dialogue and critical reflection in order to help individuals reclaim their dignity and value as subjects, thereby fostering processes of humanization and liberation. Through such educational practices, learners can develop a stronger sense of self-worth and agency, which are essential for psychological well-being and personal empowerment.

Finally, with regard to human flourishing, there are grave reasons for concern about the prevalence and etiology of mental illness. Unipolar depression, for example, strikes many individuals annually and recurrently throughout life. Upwards of one-half of adults may experience a serious mental illness in their lifetime (Keyes, 2002). Both Keyes (2002) and Seligman (2011) contend that mental health should not be understood merely as the absence of mental illness. Rather, it encompasses the realization of human potential, active social participation, and the construction of a meaningful life. From this perspective, Freire’s critical aesthetics—with its emphasis on creative expression, critical reflection, and social praxis—contributes not only to the enhancement of psychological well-being but also to individuals’ active engagement in social transformation and public life. Therefore, significant theoretical connections exist between Freire’s critical aesthetics and contemporary theories of mental health. At their core, both seek to promote human dignity, agency, social participation, and the holistic flourishing of individuals and communities.

Therefore, from the perspective of theoretical development, Freire’s critical aesthetics can be systematically connected to contemporary theories of mental health through the following theoretical pathway: humanization → recognition → autonomy and relatedness → psychological well-being → human flourishing. This framework illustrates how Freire’s educational philosophy extends beyond pedagogical concerns and contributes to a deeper understanding of the conditions that foster mental health and human development. By clarifying the relationships among these concepts, the present study establishes a more coherent and systematic connection between Freire’s critical aesthetics and contemporary mental health theories, thereby.

strengthening the manuscript’s theoretical integration and internal coherence.

### 5.2 Freire’s critical aesthetics in dialogue with contemporary theoretical perspectives

#### 5.2.1 Dialogue between Freire’s critical aesthetics and positive psychology

Positive psychology has been increasingly applied in education, health, workplace and organisational settings, and coaching and personal development. Positive psychology is known as the new branch of psychology that systematically studies conditions that contribute to the optimal functioning of individuals, groups, and institutions so that a valuable, flourishing and meaningful life is worth living (Chim et al., 2024; Seligman and Csikszentmihalyi, 2000; Herman, 1992).

Freire’s critical aesthetics and Positive Psychology both focus on human growth, well-being, and the realization of human potential, emphasizing individuals’ capacity for continuous development and self-transcendence. However, significant differences exist between the two perspectives in terms of their theoretical orientations and primary concerns. Positive Psychology primarily examines the psychological factors that promote individual well-being and human flourishing, highlighting the importance of positive emotions, meaning in life, interpersonal relationships, and personal strengths for mental health. In contrast, Freire does not regard human development merely as an individual psychological process; rather, he situates it within specific social, cultural, and political contexts. Freire argues that structural conditions such as oppression, marginalization, and dehumanization not only restrict individuals’ freedom and dignity but also hinder their development as autonomous and reflective subjects. Consequently, genuine human flourishing involves more than the enhancement of individual happiness. It also requires the cultivation of critical consciousness and engagement in processes of social transformation through dialogue and praxis (Freire, 1994, 2000; Keyes, 2002; Seligman, 2011).

Furthermore, the concepts of human flourishing and the construction of meaning in life emphasized in Positive Psychology share several commonalities with Freire’s notion of humanization. Both perspectives maintain that education should foster the realization of human potential and support individuals in constructing meaningful lives. However, whereas Positive Psychology tends to focus on individuals’ internal resources and psychological strengths, Freire further argues that the achievement of human flourishing must also address issues of social justice, democratic participation, and structural inequality. Accordingly, Positive Psychology contributes to understanding the psychological mechanisms underlying the development of psychological well-being, while Freire’s critical aesthetics complements this perspective by highlighting the importance of social critique and emancipatory praxis. Through a dialogue between these two perspectives, a more comprehensive understanding of mental health and human flourishing can be achieved—one that not only examines how individuals attain happiness and well-being but also considers how societies can create conditions that enable all people to realize their dignity, agency, and full development (Freire, 2000; Keyes, 2002; Seligman, 2011; VanderWeele, 2017).

#### 5.2.2 The connection between Freire’s critical aesthetics and recognition theory

Honneth builds and expands his theory, drawing from Hegelian philosophy as well as the psychological and anthropological ideas of contemporary Hegelian thinkers like George Herbert Mead. With a preference for the Jena works of Hegel, namely the System of Ethical Life and Realphilosophie, Honneth adopts Hegelian misconceptions and criticism of Kantian moral philosophy, presenting an ambivalent approach toward Kant’s contribution. Through this theoretical synthesis, Honneth constructs a normative framework that explains how individual identity and social integration are constituted through relations of recognition (Honneth, 1996, 2014; Salman, 2024).

A meaningful theoretical dialogue can be established between Freire’s critical aesthetics and Recognition Theory, as both perspectives are concerned with human dignity, subjectivity, and human development. They share the assumption that individuals do not develop in isolation; rather, self-understanding and personal growth emerge through relationships with others and participation in social life. In particular, the Recognition Theory developed by Axel Honneth provides an important theoretical framework for understanding how Freire’s critical aesthetics may contribute to psychological well-being and human flourishing. According to Honneth (1996), the development of self-identity and a healthy personality depends fundamentally on experiences of recognition from others. He identifies three spheres of recognition: love, rights, and social esteem. Through loving relationships, individuals develop self-confidence; through the recognition of rights, they cultivate self-respect; and through social esteem, they acquire a sense of self-worth. Conversely, experiences of exclusion, discrimination, marginalization, or disrespect may undermine individuals’ self-confidence, self-respect, and psychological well-being. This perspective strongly resonates with Freire’s analysis of oppression and dehumanization. Freire (2000) argues that oppression is not merely an economic or political phenomenon; it is also a process that denies individuals their dignity, agency, and status as subjects. When people are treated as passive recipients rather than active participants capable of thinking, dialogue, and action, their process of humanization is constrained (Freire, 2000). From the perspective of Recognition Theory, such conditions may be understood as forms of misrecognition, which hinder the development of self-identity and subjectivity. Furthermore, Freire’s emphasis on dialogue can be interpreted as an educational practice that promotes recognition. For Freire, authentic dialogue is grounded in love, humility, trust, and hope (Freire, 2000). Through dialogue, individuals cease to regard one another as objects and instead recognize each other as subjects possessing dignity and value. This process of mutual recognition not only facilitates the co-construction of knowledge but also nurtures self-confidence, self-respect, and social participation. Consequently, Freire’s critical aesthetics extends beyond aesthetic experience or artistic expression and becomes a social practice aimed at fostering subject formation and humanization.

For several decades, scholars in the fields of philosophy of medicine and philosophy of psychiatry have sought to clarify the boundaries of illness by developing scientific and conceptual frameworks for defining what constitutes a disorder. More recently, researchers have increasingly argued that mental health should not be understood merely as the absence of mental illness or the correction of dysfunctional symptoms. Rather, mental health encompasses broader concerns related to human agency, meaning, dignity, social participation, and the conditions that enable individuals to flourish (Rashed, 2021). From this perspective, Recognition Theory provides an important conceptual bridge between Paulo Freire’s critical aesthetics and psychological well-being. Research suggests that individuals who experience respect, acceptance, and social recognition are more likely to develop positive self-concepts, greater resilience, and higher levels of overall well-being (Honneth, 1996; Keyes, 2002; Keyes, 2007; Rashed, 2021). Conversely, experiences of exclusion, marginalization, or misrecognition may contribute to psychological distress and undermine individuals’ sense of self-worth and social belonging.

This perspective strongly resonates with Freire’s analysis of oppression and humanization. Freire argues that individuals become fully human through dialogue, participation, and critical engagement with social reality. Therefore, mental health should not be understood merely as adaptation to existing social conditions; rather, it should be viewed as the capacity to develop subjectivity, agency, hope, and dignity through meaningful participation in social life. From this standpoint, Freire’s critical aesthetics expands contemporary understandings of mental health. It emphasizes the importance of recognition, humanization, and transformative praxis in promoting psychological well-being and human flourishing. Accordingly, mental health is not simply an individual psychological state but a humanized mode of existence achieved through dialogue, participation, and social praxis.

Accordingly, Freire’s emphasis on humanizing education, dialogical relationships, and the development of critical consciousness contributes not only to social emancipation but also to psychological health and human flourishing. In summary, Recognition Theory helps explain how Freire’s critical aesthetics promotes subjectivity and human development through processes of humanization and dialogue. At the same time, Freire extends Recognition Theory by arguing that recognition should not remain confined to interpersonal relationships but should also involve educational and social efforts to challenge oppressive structures and create conditions that enable all individuals to realize their dignity and full potential. Thus, the two perspectives form a complementary theoretical relationship, jointly emphasizing the importance of dignity, social participation, and human flourishing.

#### 5.2.3 Operationalization of key concepts

Drawing upon the critical aesthetics of Paulo Freire, this framework proposes that *humanization* serves as the foundational condition for individual and social development. Through authentic *dialogue*, individuals engage in mutual learning and reflection, enabling the emergence of *recognition*, whereby people acknowledge both their own humanity and that of others. Such recognition strengthens individuals’ sense of *subjectivity* and *self-worth*, fostering agency, dignity, and self-respect. As individuals develop a more positive and empowered self-understanding, they are more likely to experience enhanced *psychological well-being*, characterized by meaning, resilience, and emotional health. Ultimately, these processes contribute to *human flourishing*, understood as the realization of human potential, personal fulfillment, and active participation in a more just and democratic society (Freire, 1994, 1997, 2000; Honneth, 1996; Keyes, 2002; Seligman, 2011; VanderWeele, 2017).

## 6 Conclusion

In his later years, Paulo Freire may well be regarded as one of the most significant educational thinkers of the latter half of the twentieth century. He was also a political activist and a passionate reformer. It would not be an exaggeration to describe Freire’s influence as global in scope. Yet, as with every human life, there is a beginning and an end. In the early morning of May 2, 1997, Freire passed away from a heart attack at the age of 76. In the days following his death, his close friend Moacir Gadotti expressed deep sorrow at the sudden loss, noting that Freire died at the height of his intellectual vitality. At the time of his passing, he had left one manuscript unfinished and numerous projects still in progress. Nevertheless, although Freire’s physical life had come to an end, his presence and his voice—resonant with passion—have left the world with enduring memories (Berthoff, 1987; Carnoy, 1997; Freire and Macedo, 1998; Gadotti, 1998; Roberts, 2000; Shih, 2020; Torres, 1998). Moreover, Freire’s writings in Brazil and Chile exerted a profound, if often intangible, influence on the oppressed. They rekindled hope for a renewed future and offered ways for individuals to pursue liberation and educate themselves (Haviland, 1973). In Paulo Freire’s conception of critical aesthetics, mental health should not be understood as mere adaptation to existing social realities. Rather, it signifies the restoration of the human being as a speaking, imagining, hoping, and meaning-making subject. From this perspective, mental health refers to the individual’s reconstitution as an autonomous and agentic subject—one who not only regains a sense of selfhood, but also embraces life with hope and engages in purposeful, transformative praxis within the world.

In summary, although Paulo Freire passed away in 1997, his ideas continue to exert a significant influence on scholars, educators, and social activists around the world. Widely recognized as one of the most influential educational thinkers of the twentieth century, Freire’s seminal work, *Pedagogy of the Oppressed*, remains one of the most frequently cited texts in the humanities and social sciences. Nevertheless, acknowledging Freire’s intellectual influence does not imply that his ideas should be accepted uncritically. Indeed, Freire himself consistently encouraged critical and autonomous thinking. Rather than expecting followers to reproduce his ideas mechanically, he emphasized the importance of recreating and reinterpreting them in response to changing historical, cultural, and social contexts. Freire explicitly stated that he did not wish to be followed or copied but rather reinvented through critical reflection and praxis. Consequently, engaging critically with Freire’s work is not a departure from his educational philosophy; rather, it is consistent with the very spirit of critical pedagogy that he advocated. In light of this perspective, the present study does not regard Freire’s critical aesthetics as a closed or definitive theoretical framework. Instead, it seeks to place Freire’s ideas in dialogue with contemporary perspectives, including Positive Psychology and Recognition Theory. Through such theoretical engagement, Freire’s concepts can be further clarified, expanded, and critically examined, thereby contributing to contemporary discussions of mental health, psychological well-being, and human flourishing (José Muller, 2026; Freire, 1994, 1997, 2000; Honneth, 1996; Keyes, 2002; Seligman, 2011; VanderWeele, 2017).

The article entitled *The Implications of Paulo Freire’s Critical Aesthetics for Individual Mental Health* addresses a significant and timely research topic, particularly given its interdisciplinary engagement with psychology, education, and critical theory. The intersection of these fields highlights the study’s scholarly relevance and potential contribution to contemporary academic discourse.

## Data Availability

The original contributions presented in the study are included in the article/supplementary material, further inquiries can be directed to the corresponding author.
